# EpiCollect+: linking smartphones to web applications for complex data collection projects

**DOI:** 10.12688/f1000research.4702.1

**Published:** 2014-08-20

**Authors:** David M. Aanensen, Derek M. Huntley, Mirko Menegazzo, Chris I. Powell, Brian G. Spratt

**Affiliations:** 1 Department of Infectious Disease Epidemiology, Imperial College London, London, W2 1PG, UK; 2 Centre for Bioinformatics, Imperial College London, London, SW7 2AZ, UK

## Abstract

Previously, we have described the development of the generic mobile phone data gathering tool, EpiCollect, and an associated web application, providing two-way communication between multiple data gatherers and a project database. This software only allows data collection on the phone using a single questionnaire form that is tailored to the needs of the user (including a single GPS point and photo per entry), whereas many applications require a more complex structure, allowing users to link a series of forms in a linear or branching hierarchy, along with the addition of any number of media types accessible from smartphones and/or tablet devices (e.g., GPS, photos, videos, sound clips and barcode scanning). A much enhanced version of EpiCollect has been developed (EpiCollect+). The individual data collection forms in EpiCollect+ provide more design complexity than the single form used in EpiCollect, and the software allows the generation of complex data collection projects through the ability to link many forms together in a linear (or branching) hierarchy. Furthermore, EpiCollect+ allows the collection of multiple media types as well as standard text fields, increased data validation and form logic. The entire process of setting up a complex mobile phone data collection project to the specification of a user (project and form definitions) can be undertaken at the EpiCollect+ website using a simple ‘drag and drop’ procedure, with visualisation of the data gathered using Google Maps and charts at the project website. EpiCollect+ is suitable for situations where multiple users transmit complex data by mobile phone (or other Android devices) to a single project web database and is already being used for a range of field projects, particularly public health projects in sub-Saharan Africa. However, many uses can be envisaged from education, ecology and epidemiology to citizen science.

## Introduction

The increasing availability and decreasing cost of mobile devices running the open-source Android operating system ^1^, in conjunction with greater geographic penetration of mobile data networks and concurrent increase in bandwidth ^2^, provides ever-increasing opportunities for the use of such devices in many kinds of data gathering projects. Existing initiatives within this area broadly fall into two groups. Firstly, those that focus on standard mobile devices and take advantage of standard mobile networks for the transmission of data (e.g., using SMS to pass text messages, as in RapidSMS ^3^ or richer form-based data in EpiSurveyor (now Magpi) ^4^) and, secondly, those that take advantage of mobile data networks to enable the passing of data via the web (e.g., ODK ^5^ and EpiCollect ^6^).

Common to all initiatives is the definition of the data to be gathered as a ‘form’ which details the questions presented to a user, with the method of form definition varying between initiatives. SMS-based initiatives generally utilise coded responses to compress the answers to questions into short strings, which can be transmitted via SMS. At the other end of the spectrum, and more generally with respect to the initiatives that use mobile data networks for data transfer, more complex single forms can be defined using standard vocabularies (usually eXtensible Markup Language, XML), which are loaded onto a phone, with data gathered and sent centrally for further analysis via the web. The complexity of setup, form definition and functionality available to an end user varies between initiatives; however, common to all is the definition of only a single form per project.

EpiCollect was developed to provide a simple and intuitive method for complete online project creation, data storage and visualisation of aggregated data captured using smartphones based on a single form. Since release, EpiCollect has been used for a broad range of data collection projects using Android smartphones, and the data collection form for a specific project and an associated web application can be generated online by a simple process at the EpiCollect website ( www.epicollect.net). Despite its continuing utility, and ease of setup, many data collection projects require considerably more complexity than a single form. For example, to use mobile phones to collect information about the vaccination status of children in schools in a region of a developing country, we would need a form for each school in the area and, linked to each school, a form for each class in the school and, linked to each class, a form for each child in the class. This hierarchy of forms allows data to be collected in a ‘one-to-many’ fashion as required by the project. Additionally, we may need branches in this linear hierarchy of forms so that, if a question on a form is answered ‘yes’, an additional form that needs to be completed one or more times appears on the phone as a branch link.

A greatly enhanced version of EpiCollect (EpiCollect+) has therefore been developed that provides the ability to produce individual data collection forms that are more complex than the single form used in the original version (which is now referred to as EpiCollect or EpiCollect v1), and to link multiple forms together in a linear or branching hierarchy. These new features of EpiCollect+ make it a very versatile tool for data collection via mobile phones. The EpiCollect (and EpiCollect+) app has been developed for any device running the Android operating system (e.g. mobile phone or tablet); however, for convenience we will henceforth refer to ‘mobile phone(s)’.

## EpiCollect v1 – a framework for simple mobile data gathering projects

**Figure 1.**
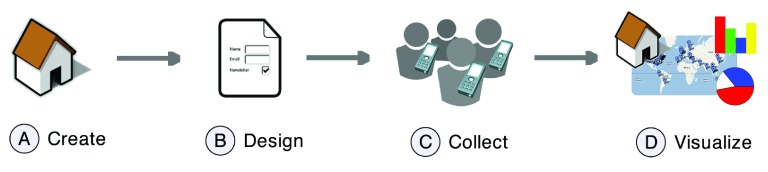
The EpiCollect workflow. Website creation is undertaken at www.epicollect.net ( **A**), followed by online form design ( **B**). A project is loaded onto one or more phones and, following data gathering and synchronisation ( **C**), data are visualised using Google Maps and charts at the project website ( **D**). Data can also be entered or downloaded directly via the project website.

We have previously detailed the overall framework for mobile data gathering using EpiCollect ^6^ and here we briefly detail the online process for project definition and data visualisation that is now available at www.epicollect.net ( Figure 1).

EpiCollect v1 projects are hosted using Google’s cloud computing platform AppEngine (GAE) ^7^ primarily for two reasons. Firstly, GAE provides automatic scalability and, secondly, we can readily use Google Accounts authentication (passwords) for assigning ownership to a particular project. Although GAE is used extensively for data storage and application provision, EpiCollect v1 software and documentation are provided to allow project setup on other servers with data sent/retrieved from the mobile app to/from a database set up outside of GAE.

All methods detailed here are generic and applicable to many kinds of projects. However, for demonstrating how a simple EpiCollect v1 project is setup online using GAE, we describe a hypothetical single form data gathering project where we wish to undertake a survey of schools. Initially, we wish to know five things – the name of each school, the type of school (state, independent etc.), whether the school is single-sex or mixed, and the number of teachers and pupils. Furthermore, we wish to capture an image of each school and record its geographic location.

**A) Create.** Naming a project and creating a permanent project homepage.

The ‘Create project’ link at www.epicollect.net generates a prompt for entering a new project name – in this case ‘schoolsdemo’. Following input (and a check to make sure the name is unique), a permanent URL (the project homepage) is generated that includes the project name (as follows):

http://www.epicollect.net/project.html?name=schoolsdemo

**B) Design.** Specifying the survey questions using a ‘drag and drop’ form builder.

**Figure 2.**
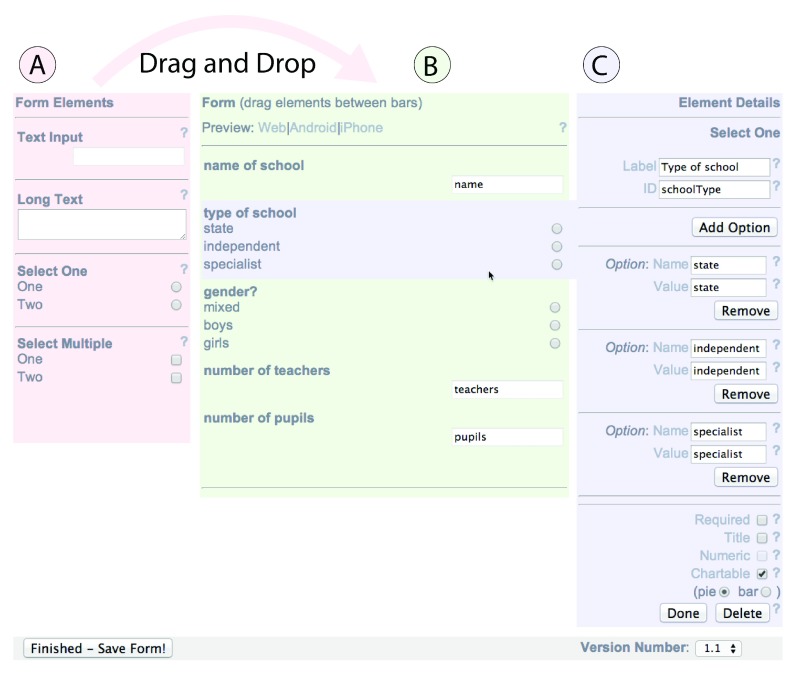
Creating a form using the EpiCollect v1 ‘drag and drop’ interface. Form elements available are listed on the left ( **A**); any number can be dragged to the central section to build up a form ( **B**). For each form element chosen, details are entered on the right ( **C**) including the text presented to a user and possible choices should a pre-defined list of answers be required.

A data collection form detailing the questions to be asked of each individual participating in the survey is now generated. The project owner is first prompted to login to epicollect.net using a Gmail account and, henceforth, only this Gmail account holder can amend the project form and edit the project homepage. Once logged in, the project owner is presented with a ‘drag and drop’ form builder, which provides a simple and intuitive way to define the questions presented to a user and consists of three sections ( Figure 2). A list of the types of available Form Elements are shown ( Figure 2A), and these are ‘dragged’ to a blank form in order to build up the required questionnaire ( Figure 2B). Four types of text field are available – ‘Text input’ where a user enters free text, ‘Long text’ which allows a larger number of text characters than the ‘Text input’ option, ‘Select One’ which is equivalent to a dropdown list where choices are defined and a user can select ONLY one answer, and ‘Select Multiple’ which is equivalent to a checkbox list where choices are pre-defined and a user can select one or more answer(s).

When dragging a particular form element onto the form, a user must define the element details that specify the particulars about an instance of that element ( Figure 2C). These include the ‘Label’ (which specifies the text displayed to a user answering the questionnaire) and the ‘ID’ (a unique name given to a particular data field and used as a reference when storing the data - the equivalent of a column heading in a spreadsheet or database table). For multiple choice questions (Select One and Select Multiple), the available choices can be defined at this point where the ‘Name’ specifies what is shown to the user and the ‘Value’ indicates what is stored in the database when the particular option is chosen by a user. Validation for a particular field can be set as ‘required’ or ‘numerical’ or both, to denote that the question MUST be answered and/or must only contain an integer. Finally, for ‘Select One’ (dropdown) and ‘Select Multiple’ (checkbox) elements, charting can be enabled, so that a pie or bar chart showing the distribution of the answers of users will be shown when the data submitted by mobile phone(s) are viewed at the project website. There is no limit on the number and type of questions that can be specified in a form, although the number of chartable fields is currently limited to three. In this way, forms containing many questions can be built up easily on-line and tailored to the data gathering needs of the project owner. On completion of a form, the project is ready to be loaded onto one or more mobile phones.

**C) Collect.** Loading a project onto a mobile phone(s) and collecting data.

The EpiCollect v1 mobile app is first installed on each Android phone to be used in the survey (via Google Play or direct download from the EpiCollect website). From the mobile app’s homescreen, entering the project name in the ‘Load Project’ menu option (e.g., ‘schoolsdemo’) requests the project definition file from GAE. Once loaded, three main options are now available from the homescreen - ‘New entry’, ‘List entries’ and ‘Display map’. ‘New entry’ presents the data collection screen. Each new entry is assigned a unique ID and automatically date and time stamped. A location (if GPS or wireless is enabled on the phone), and a photo (captured using the phone’s camera), can be attached to an entry and then the questions defined in the survey form are answered. Each completed entry is stored in the phone’s database, allowing many entries to be collected on one handset (only limited by memory). Synchronising data to the project website is achieved by listing the entries and tapping ‘synchronise’, which sends a copy of the survey data gathered on the mobile phone to GAE, which can then be viewed at the project website along with entries already submitted from other phones being used in the project. Multiple projects can be loaded on a single phone and the project to be undertaken can be switched from a dropdown list on the mobile app’s homescreen.

Data connectivity (e.g., 2/3/4G or wireless) is only required when initially loading the project into the app and when synchronising the data with the project database. Importantly, it is NOT required for entering data (GPS does not require data networks), allowing data gathering in remote areas with later synchronisation when data connectivity is available. Furthermore, data from GAE can be requested to the mobile app so that data previously collected by the user, and/or other data collectors, can be viewed on any handset being used in the project.

**D) Visualise.** Viewing data at the project homepage.

**Figure 3.**
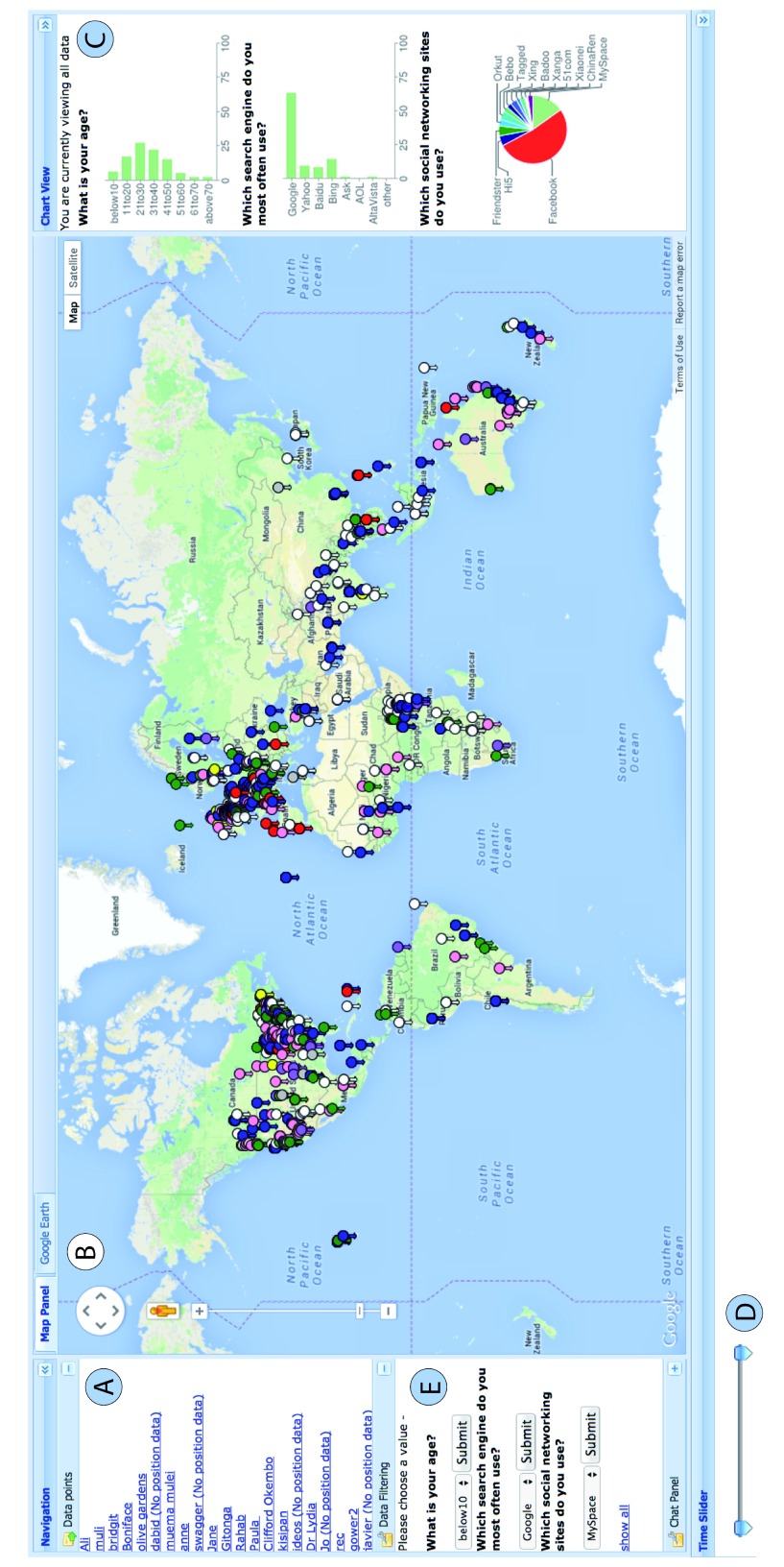
The EpiCollect v1 mapping interface. Data shown are from a demo-project, which comes preloaded with the EpiCollect v1 mobile application. All data points for a project are listed at the top left ( **A**) and their position (if recorded) is plotted on a Google Map ( **B**). Any fields that were specified to be charted when designing a form are displayed in the Chart View ( **C**). A temporal slider allows the data collected within a specified time period to be displayed ( **D**). Data can be filtered based on any ‘Select one’ or ‘Select multiple’ field, allowing the display of only those entries with the specified value(s) ( **E**).

The data collected from one or more phones during the survey can be viewed via the project homepage in two ways. Firstly, data can be listed in a tabular view, with columns based on the defined form fields, and all data can be downloaded in a number of formats (CSV/XML) for use and further interpretation outside of EpiCollect. Secondly, data can be viewed using a generic mapping interface ( Figure 3). Briefly, all entries where location data have been captured (latitude/longitude via either GPS or wireless) are listed ( Figure 3A) and can be viewed on a Google Map (or within Google Earth). The Google Map interface ( Figure 3B) includes charts (pie and/or bar) for displaying data from fields defined as ‘chartable’ when building the project form ( Figure 3C), and a temporal ‘slider’ allows only those points gathered within a particular time period to be viewed ( Figure 3D). Data that are chartable can also be filtered based on choosing a particular ‘answer’ to a question that was presented to the users, so that only those entries where this answer is given are shown on the map ( Figure 3E).

## Using XML to define projects

Within EpiCollect we have focussed on complete generality allowing the design of any kind of project that would benefit from the collection of questionnaires along with location and photographic data. This generality is achieved by defining a project description using XML.

For EpiCollect v1, we defined a set of XML tags (a vocabulary; ^8^) that allow all elements of project description to be defined in a single document. Within the online interface, the XML details are hidden from the user. However, in brief, when defining a form, the ‘drag and drop’ form builder produces an XML description of the form, including the name of the project. This XML file is then utilised both on the GAE server (for production of the generic map interface/tabular view when data is viewed) and within the mobile app when a project is loaded, to indicate the questions to be presented to a user and the location of the GAE server (tagged by project name) to send/retrieve data to/from.

We utilise XML for a number of reasons. Firstly, it allows a standard and flexible vocabulary for defining projects; secondly, it allows users to host and undertake their own data gathering projects using their own databases and servers. By default, when loading a project on the EpiCollect mobile app, a request is sent to GAE for retrieval of the corresponding project description (in XML). However, XML can be loaded from any web location by typing in a fully resolved URL. Thus, by defining a project in XML (including server location and scripts for handling data exchange), and hosting on a local server, users can utilise the mobile app for data gathering and retrieve and store all data on their own servers. Further instructions are available in the developer’s section at epicollect.net.

Because XML is extensible, we can expand our vocabulary as and when required. As long as the software that utilises the XML ‘understands’ the vocabulary, tags can be defined which address greater complexity. Expansion of this principle (additional tags, interpreted by server and mobile software) forms the basis of progressing from EpiCollect v1 to EpiCollect+.

## From EpiCollect to EpiCollect+

While EpiCollect v1 may prove adequate for many types of projects, in other cases, a greater control over the flow of questions becomes necessary and projects may require the collection of multiple linked forms within a single project. Similarly, the limit (and requirement) for the collection of a single image and a single GPS may be restrictive and does not take advantage of other media types available to phones running the Android operating system. For example, the camera can be used to capture short videos, or for the extraction of data encoded in barcodes, the microphone can be used to capture sound clips. Moreover, it is possible, through Bluetooth bridges, to connect to other ‘sensors’ for the acquisition of data additional to those provided from mobile phones (e.g., Arduino ^9^).

We have therefore developed EpiCollect+, which uses a greatly enhanced XML vocabulary for project description than that used for EpiCollect v1, allowing multiple text and/or media fields, multiple linked forms, and increased validation and control over form logic, to be specified easily. Furthermore, as described for EpiCollect v1, we include a ‘drag and drop’ interface for EpiCollect+ project definition, providing a complete online solution to complex project definition and data gathering.

**Figure 4.**
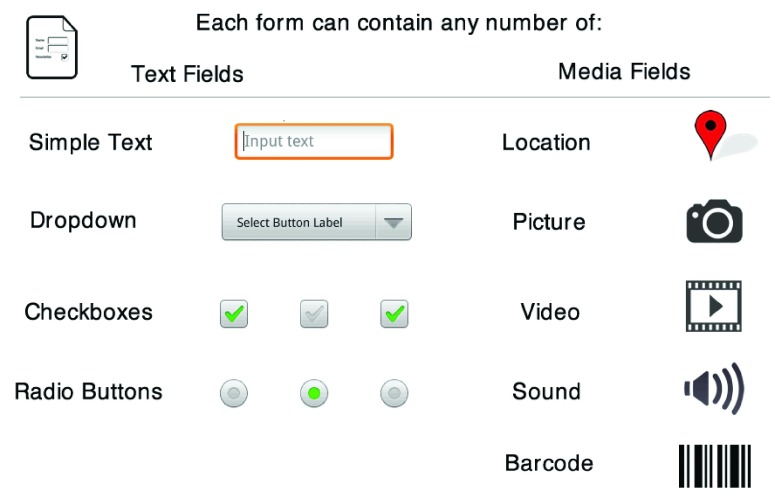
Form fields available within EpiCollect+. Text fields mirror those available in EpiCollect v1 and media fields include video clips, sound clips and barcode scanning. Any number (0, 1 or many) of any of these fields can be included in a form.

In brief, the EpiCollect+ XML description allows the definition of projects that contain one or more linked data forms (linked either hierarchically, in a one-to-many fashion, or branched) and each form can contain any number of the standard ‘text’ and ‘media’ fields ( Figure 4). We describe the enhanced features of EpiCollect+ by first outlining the increased complexity available for the individual data collection forms, and then the hierarchical and branch linking of multiple forms.

## Text fields

As well as the text fields described for EpiCollect v1 (‘Select one’ fields are henceforth referred to as ‘Dropdowns’ and ‘Select multiple’ as ‘Checkboxes’), EpiCollect+ allows the use of radio buttons which are conceptually similar to a dropdown list, in that choices are predefined and a user is allowed only one choice; however all options are displayed on the phone’s screen at one time. Within an individual form, any number of such fields can be specified and are presented to a user in the order defined. However, defining form logic (e.g., setting skip patterns) and validation of the data entered, allow greater control over the data entered (see below – Form data logic and validation).

## Media fields

XML tags have been specified that allow the capture of a number of additional media types to those available in EpiCollect v1:

**Location**: The phone’s position obtained using built-in GPS or via network or wireless positioning. The latitude, longitude, altitude and accuracy (in metres) are recorded.

**Image**: An image from the phone’s picture library or an image captured using the phone’s camera.

**Video**: A video clip, captured using the phone’s camera.

**Audio**: A sound clip, captured using the phone’s microphone.

**Barcode**: Extraction of data encoded within a 2D barcode, using the phone’s camera, is entered into a text field within the form (utilising either Google’s built-in barcode scanner within recent versions of Android or the ZXing library ^10^, which users are prompted to install if no barcode reader is detected).

Any number of each media type (0, 1 or more) can be defined within a form and all data are synchronised to the server and available for download/viewing.

## Data logic and validation

EpiCollect+ provides control over the flow of questions within a form using the following methods:

**Form logic**: This allows the definition of questions to ‘jump to’, should a particular answer be given to a particular question. For example, if the answer to Q1 is A, jump to Q5; for all other choices continue to Q2. Multiple clauses within a jump can be defined to allow greater flexibility. For example, if the answer to Q1 is A or C, jump to Q5, or if the answer is B, jump to Q7, else continue to Q2. A common example where jumps are used is to record a value for a question with defined choices (i.e. dropdowns, radio buttons) where one of the choices is ‘other’. Thus, if Q1 is ‘What is your favourite colour?’, with the options ‘blue’, ‘red’, ‘green’ and ‘other’, a jump to Q3 could be used if one of the pre-defined colours was selected. If ‘other’ was chosen the user would proceed to Q2 (containing a simple text box), where they would be asked to enter their favourite colour (the ‘other’ colour that was not in the initial list) before proceeding to Q3.

**Data validation**: Data validation within the description of a form allows the setting of rules that attempt to minimise the opportunity for data entry errors or entry of incorrect data types. Free text is open to the potential for spurious data input and a number of methods are provided for specifying data validation. These include requiring fields and adherence to a particular data type; e.g., integer only, dates and times with one of a number of specified formats, such as dd/mm/yyyy or mm:ss, and minimum and/or maximum accepted values for numeric fields allowing the definition of ranges. Furthermore, date and time fields can take advantage of graphical ‘widgets’ to provide scrollwheels for entering dates.

Regular expression pattern-matching is also fully implemented within EpiCollect+, allowing the definition of a particular string pattern for matching against a user’s input, with warnings should the data entered conflict with the pattern specified. Such validation is suited to free text but can also be applied to other field types. For example, regular expressions can be used in conjunction with barcodes. Should a barcode be known to encode data starting with a particular series of characters, this can be specified using a regular expression and a warning returned should the wrong type of barcode be scanned. Any text field (and also barcode fields) can also be flagged for ‘double entry’ and when users are presented with the question (or request to scan the barcode), they are prompted to enter the data twice. Cross-checking ensures that both entries are identical before proceeding to the next question (as commonly used within web forms for passwords that have to be entered twice and are cross-checked).

## Linking forms

In EpiCollect+ multiple single forms can be linked together within a project in two ways – hierarchical and branching.

**Hierarchical linking**: The schools project mentioned above is used to demonstrate the general principles of form linking available in EpiCollect+. Within this schools project, we intend to survey schools from across a defined area and to record details about each school. However, in addition, we now wish to collect information about each class (class size, class teacher etc.), and about each pupil in each class. Furthermore, for each pupil, we wish to collect two media types. Firstly, a short audio clip of the pupil pronouncing their own name and, secondly, we wish to take a blood sample, which will be processed by a laboratory to obtain their blood group, with these data subsequently being added to the survey. For the audio, we will use the phone’s microphone to record a short clip and, for the blood sample, we will use pre-printed barcodes and ‘scan’ a barcode, associating the code to the entry before sticking the printed barcode to a blood sample tube.

**Figure 5.**
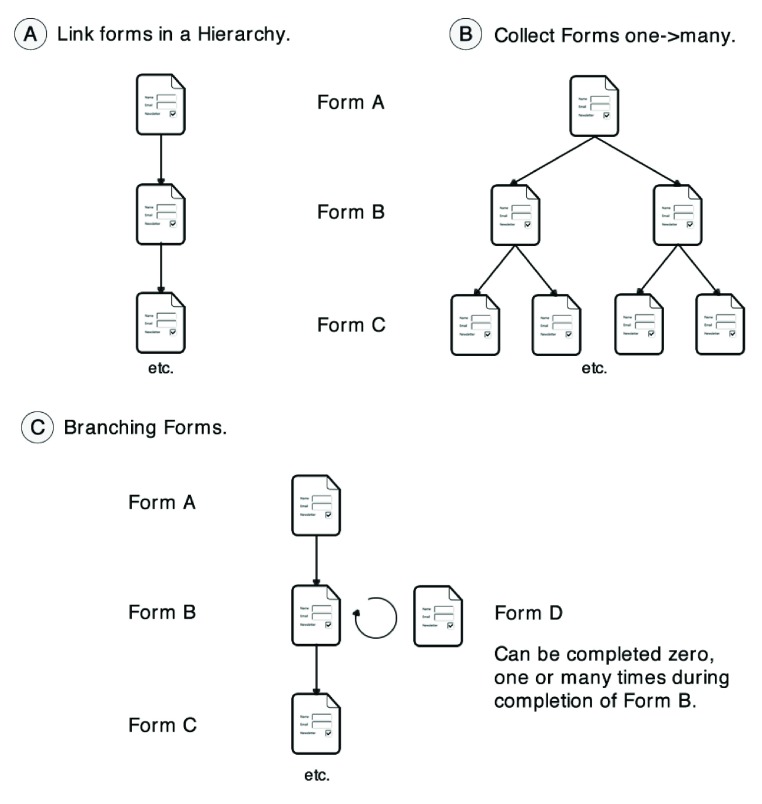
Linking forms. Forms can be linked hierarchically ( **A**), allowing a ‘one-to-many’ relationship between the data gathered ( **B**). Forms can also be linked as a branch ( **C**) allowing, at any point within a form, ‘branching’ out to fill in one or more entries for a branch form.

To undertake data gathering, we could design a single form that, for each pupil, includes details about their class and their school, but this would lead to vast repetition of data entry, and there is a natural hierarchy within the project that lends itself to three forms being used in a ‘one-to-many’ fashion. Each school has many classes and each class has many pupils. If we define this hierarchy from top to bottom as school->class->pupil, we can design a single form for each of these three levels, and link them together so that the one-to-many hierarchy is preserved. Within EpiCollect+ this is achieved by adding a new form and defining a ‘key’ field that is used to link data in one level of the hierarchy to the level above and/or below ( Figure 5A). Each level is defined as a single form (including any number of text and media fields) and linked by key fields, allowing data gathering to occur in the one-to-many fashion ( Figure 5B). A key field should contain data that are unique to each instance of a form. For example, within the schools project, we could define ‘school name’ as the key field in the school form (each school will have a unique name). This is linked to the class form so that all class forms contain the name of the school they are associated with. Similarly, we could define ‘class name’ (each class will have a unique name) as the key field in the class form which is included in all pupil forms, linking specific pupils to a class within a school.

**Branch linking**: A second method of linking forms is provided by the branch form type ( Figure 5C). Within the schools project we may wish to record, within each class form, the number of absences taken by a teacher during the previous academic year and, for each absence, to capture details specific to each absence (e.g., reason for absence, number of days etc). Initially, the number of absences per teacher is unknown and may require no additional forms (a teacher has not been absent) or many ‘absence’ forms (a teacher has been absent more than once). This uncertainty in the number of forms required for each teacher precludes inclusion in a hierarchy as this would require at least one entry per teacher and, therefore, does not logically fall into the one-to-many linear hierarchy. To address these kinds of extra data forms, we allow the definition of a ‘branch form’. A question within the initial form (e.g., ‘Please record absences’) causes the branch to be triggered and presented to the user. In this fashion, when a user fills in details of each class there is a question about the number of absences of their teacher. The response to this question triggers the ‘absence’ branch form (if required) and the required number can be filled in before continuing with the next question on the class form. If there are no absences, the class form continues automatically to the next question. Branch forms can be defined at any point within any form within a hierarchy and, furthermore, multiple branches can be defined within a single form.

Defining a project in this fashion means that initially, we simply describe the fields (text and/or media) for each type of form, and link them together to provide the overall hierarchy that is required.

## Defining keys and synchronising data with the central database

As in EpiCollect v1, the data collected on each mobile phone are synchronised with the central server. However, in EpiCollect+, data are sent sequentially from the highest level in the hierarchy downwards to preserve the integrity between ‘keys’ in forms at all levels. An entry from a form lower in the hierarchy must have an entry ‘above’ it for the entry to be successfully entered and synchronised.

When designing a form, a user can choose to define their ‘key’ field or we offer the ability to automatically generate a key, which can be displayed or hidden from the user on the form. The auto-generated key consists of the phone’s universal unique identifier, concatenated with the exact time the entry is taken in milliseconds. This precludes the possibility of data being entered more than once.

## Undertaking a project with EpiCollect+

**Figure 6.**
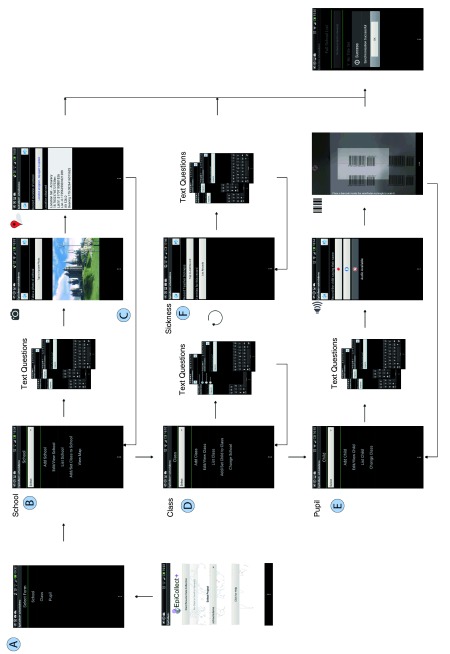
Workflow for entering data into the Schools project. Loading the Schools project lists three forms in the ‘Select Form’ menu in the order ‘School’, ‘Class’ and ‘Pupil’ ( **A**). Selecting the top level form (School) presents the School menu ( **B**), containing options for viewing or adding data. ‘Add School’ presents the user with the questions specified (text and/or media) allowing single or multiple school entries to be added ( **C**). After completion of an entry, further schools can be added or a ‘Class’ (representing the second level in the hierarchy) can be added to a particular school ( **D**). Similarly, after completion of a Class entry a user may choose to add another Class or drop to the third level of the hierarchy and add one or more Pupils to a particular Class ( **E**). The presence of a branch ‘Sickness’ form in the Class form can be utilised to add any number of Sickness forms to a particular Class as described in the text ( **F**). Synching can be undertaken at any point within the form hierarchy.

As in EpiCollect v1, all elements of project design in EpiCollect+ are undertaken online using a ‘drag and drop’ form builder and the project is loaded onto one or more phones. When starting data collection, forms are listed in the order defined in the hierarchy and must be entered in this fashion. In the schools project, the forms are listed in the order ‘school’, ‘class’, ‘pupil’ (See Figure 6). Once the top level ‘school’ form is filled in, a menu is shown which offers three choices. 1) ‘Add another school’, allowing a new entry to be added. 2) ‘List/Sync schools’, which allows all school entries to be viewed and synchronised with the central server. 3) ‘Add class to school’, which allows the user to fill in a form immediately below in the hierarchy, which will be associated with the school through the school forms key field. Each level of the linear hierarchy works in this way – i.e., on completion of a form entry, users are prompted to add entries in the level below in the hierarchy.

When completing a form on a mobile phone, text fields are completed via the keyboard and, for media fields, the user is prompted to record the media specified. For example, for any location field (i.e., GPS), the user is prompted to ‘tap-to-set location’ and if a GPS or wireless signal is available the screen will be updated with location details (latitude/longitude and accuracy). For picture or video fields, the phone’s camera is launched (or the user can select from the phone’s library of images/videos). For audio clips, audio controls allow ‘stop/start’ for recording a short clip. For barcode scans, the phone’s camera is launched, allowing the user to point the camera at a barcode and, once recognised, the data encoded are extracted and placed in a textbox on screen.

Synchronising data can be undertaken either after each entry is completed or all at once from the app’s homescreen. All textual data, from all levels in a project hierarchy, are synchronised in this fashion. For photos, a thumbnail is sent to the server by default. Due to the potentially large amount of data transfer when synchronising multiple photos, video and audio clips, we provide a menu option that allows these data to be synchronised separately. This is provided to give the user the choice of whether to send all large media data via mobile networks or wireless.

At the project homepage, as with EpiCollect v1, all data can be viewed in two ways. A list of forms, in hierarchical order, is shown allowing selection of the form entries required. A tabular view of data is shown listing all entries, allowing basic functions such as sorting/searching. For pictures, a thumbnail is present and, if clicked, the full size image is seen (if synchronised – see above). For audio and video recordings, a link is provided to download clips for listening/viewing. For any form that has a location field, a further map view is provided which, as in EpiCollect v1, places all entries on a map (clustered at lower zoom levels), which can be clicked to view further details in a pop-up window. Charts can be viewed for any dropdown, checkbox or radio button field and a temporal slider is available to view entries gathered within a particular time period. For any form, all data are available to download as comma- or tab-separated plain text files or XML which, by default, include URLs to any media fields recorded within a form.

Data collection is not reliant on a mobile phone and can be entered at any level within a project’s form hierarchy directly via the web. For data entry via the web, a similar interface is provided to that available on the phone and each question is answered in turn. For location fields, a geo-coding facility is provided that allows an address to be entered and its latitude/longitude retrieved and, for other media fields (picture, video and audio), a file can be uploaded from the user’s computer. Currently, for barcode fields, only the decrypted data can be entered via the web.

## Loading data from a project database onto mobile phones

Within any project it is possible to pre-load data from the central database onto a mobile phone at any level within a project hierarchy allowing, for example, the continuation of data gathering from mid-point within a project. For example, within the schools project, we may already know the names and details of many or all of the schools we wish to survey. We may then recruit 15 new data gatherers to aid completion of the project. Rather than re-entering the school information for the new users to subsequently add further classes/pupils, the schools project can be loaded onto their phones and, from the menu, the form level within the hierarchy to pre-load can be selected. Choosing ‘school’ would load all the data on all schools from the central server. Similarly, selecting the ‘class’ level within the hierarchy will load all school and class data. All data within and above levels in the hierarchy are loaded to preserve data integrity.

## Server software

Generic server software is provided that allows the hosting, storage and viewing of data collected using the EpiCollect+ mobile app. Briefly, the server software requires three things: a web server (e.g., Microsoft IIS or the open source Apache), a relational database (MySQL or similar) and the server-side PHP scripting language. Installation of such software should be trivial for system administrators and bundled software is available that will install and configure all three components in a single click-to-install executable (e.g. XAMPP ^11^). The only other requirement is that the server is web-accessible with either a static IP address or via a URL. This location is then specified within the phone (through a ‘settings’ option) to indicate to the mobile app where to submit and retrieve data to/from.

The same project building functionality is provided for any installed server (including the ‘drag and drop’ form builder and also the option to directly upload an XML document) and loading onto the mobile app occurs in the same fashion as a project setup at epicollect.net. When a user selects ‘load project’, and types the name of the project, the mobile app requests the project definition from the server specified. Multiple projects can be setup on a single server and each will have its own URL e.g., www.myserver.com/epicollectplus/projectname where ‘ www.myserver.com’ and ‘projectname’ indicate the server location and name of the project. Within the EpiCollect+ server software, user administration allows users to be assigned as managers/curators or users. Managers are granted full administration rights (e.g., to setup/amend projects), curators are allowed to view and enter data via the web, whereas users can only submit (or receive) data by mobile phone. Any EpiCollect+ project can be flagged as public or private and private projects require a logon to view data. User authentication methods can utilise native logon, Gmail or LDAP and https is supported.

## Discussion and future directions

Since its release in 2009, over 4000 EpiCollect v1 projects have been setup at www.epicollect.net using GAE, ranging widely in scope and subject, and v1 continues to be used within a wide variety of disciplines. For example, within Global Health it is being used for OneHealth initiatives ^12^, global wildlife disease monitoring ^13^, vaccination coverage ^14^ and within regional centres of global organisations such as the Food and Agriculture Organisation of the United Nations (FAO) ^15^).

EpiCollect+ substantially increases the flexibility of project design over that available in EpiCollect v1 and provides a generic method for users to define and contribute to complex data gathering projects of many kinds using devices running the Android operating system. EpiCollect+ has been field tested within a number of initiatives to allow the scalability of new functionality to be assessed and refined through feedback from project designers and users. For example, within public health, the Schistosomiasis Consortium for Operational Research and Evaluation (SCORE ^16^), in conjunction with the Schistosomiasis Control Initiative (SCI ^17^) have been using EpiCollect+ for the monitoring and evaluation of praziquantel treatment across multiple African countries. A 5-level hierarchically linked form structure has allowed the collection of data about multiple villages (level 1), multiple locations within villages (schools or houses – level 2) and multiple individuals within each location (level 3). Multiple specimens (blood and stool samples) are taken from each individual and tagged using barcode scanning (level 4) and data are submitted to their central database via mobile phone. Following laboratory processing of samples, data are entered via the web (level 5). One year of data collection in Kenya has resulted in ~250,000 entries being collected by teams of researchers via mobile phone (levels 1–4) and the web (level 5). Instances of the EpiCollect+ server software are setup in-country allowing the complete management of data to/from phones and via the web locally. Further examples include the gathering of human movement data to inform mathematical models of malaria transmission in Mali, Burkina Faso, Zambia and Tanzania (Marshall, J. and Ghani, A., unpublished) and for the assessment of HIV interventions in Zimbabwe (Gregson, S., unpublished).

Another use of EpiCollect v1, or EpiCollect+ for more complex projects, is within education. Many schools and universities run field courses for students studying different disciplines. For example, the University of Bath (UK) runs an annual field course focussed on basic statistical analysis for students of the biological sciences. Traditionally, students carrying out fieldwork enter data in notebooks and, following transcription onto a computer, the data are collectively analysed to investigate initial hypotheses. Over the past three years, students have undertaken data gathering utilising EpiCollect rather than on paper. Firstly, as data are stored and easily accessible via the web, temporal analysis can easily be undertaken to compare this year’s results with those from previous years, to investigate trends over time. Furthermore, the availability of the project online opens up the possibility of running the same educational project each year in different universities (or schools), and across different countries, increasing the amount of data collected and expanding the scope for data analysis, with the ability to identify both regional and temporal trends.

EpiCollect has also been used for citizen science (e.g., for the collection, by members of the public, of geo-tagged roadkill sightings to understand and inform species protection strategies in the USA ^18^). The increased functionality, and access to media fields in particular, within EpiCollect+ presents further uses within this area. For example, EpiCollect+ has recently been linked with the micro-tasking platform, http://crowdcrafting.org, allowing the processing and interpretation of data gathered via the ‘crowd’. Micro-tasking allows a simple defined task to be undertaken on large sets of data by multiple individuals providing some confidence on the majority result. One EpiCollect+ project gathers recordings of bird song, using audio clips, which are submitted to crowdcrafting for users to specify whether they can identify the particular species of bird. Further citizen science uses of EpiCollect+ are being developed as part of the www.citizencyberlab.eu project.

The use of EpiCollect v1 or EpiCollect+ should be guided by the requirements of a project. Form building using ‘drag and drop’, or by defining in XML, is more complex in EpiCollect+ than in EpiCollect v1, and this complexity is also apparent when entering data into the mobile app. The simplicity of project setup and use within EpiCollect v1 would make this the best choice for simple projects. The extension of the v1 XML vocabulary allows us to provide backwards compatibility and EpiCollect v1 projects can be loaded into the EpiCollect+ app and we will continue to support both versions. More complex data collection projects will require the much increased functionality of EpiCollect+ and the ability to run projects from the user’s own server. The ability to use ‘drag and drop’ makes the setting up of projects relatively straight forward, and the availability of bundled server software, should allow anyone with moderate knowledge of computing to set up the software and web server to run an EpiCollect+ project in-house.

We chose to develop EpiCollect+ using Android for a number of reasons. Firstly, Android is an open source platform and has a large community of developers actively contributing to its further development. At the time of the initial EpiCollect publication (2009) only two commercially available handsets running the Android OS were available. Current estimates suggest that there are now almost 400 different handsets (smartphones and tablets) from a variety of hardware manufacturers, ranging widely in price and functionality. Cheap Android devices offering full smartphone functionality are becoming more readily available in resource-poor settings and, in tandem, increases in both mobile data network coverage and speed across the globe are providing higher internet penetration, allowing EpiCollect to be used successfully for projects in most countries.

At present, EpiCollect and EpiCollect+ are only supported for Android devices. The availability of other mobile operating systems (e.g., Apple iOS, Windows Phone, and Blackberry OS) requires the production of a different codebase for each operating system, increasing development time and costs for each OS supported. A potential solution is provided by HTML5, which is the W3C standard for the production of a platform-independent software that runs entirely within an HTML5 compliant web browser ^19^. A HTML5 version of EpiCollect+, available for multiple operating systems, is currently in BETA testing.

## Software availability

### Software access

The EpiCollect+ Android app can be downloaded from Google Play.

The EpiCollect+ Server and instructions are available at: http://www.epicollect.net.

### Latest source code

The server: https://github.com/ImperialCollegeLondon/EpiCollectplus

The mobile app: https://github.com/ImperialCollegeLondon/EcPlusAndroid

### Archived source code as at the time of publication

http://dx.doi.org/10.5281/zenodo.11278 ^20^

http://dx.doi.org/10.5281/zenodo.11281 ^21^

### License

AGPLv3 Licence

## References

[1] The Android Operating System. Reference Source

[2] GSM Roaming and Coverage Maps. Reference Source

[3] RapidSMS – SMS Application Framework. Reference Source

[4] Episurveyor. Reference Source

[5] Hartung C Ankowa Y Brunette W: Open data kit: tools to build information services for developing regions. ICTD2010; Surrey, UK:2010 10.1145/2369220.2369236

[6] Aanensen DM Huntley DM Feil EJ: EpiCollect: linking smartphones to web applications for epidemiology, ecology and community data collection. *PLoS One.* 2009;4(9):e6968. 10.1371/journal.pone.0006968 19756138 PMC2735776

[7] Google App Engine. Reference Source

[8] W3C XML Specification. Reference Source

[9] Arduino, Open Source electronics prototyping platform. Reference Source

[10] Zxing, Open Source Barcode Scanning Library. Reference Source

[11] XAMPP – Open Source Apache Installation containing MySQL, PHP and Perl. Reference Source

[12] Rweyemamu MM Mmbuji P Karimuribo E: The Southern African Centre for infectious disease surveillance: a one health consortium. *Emerg Health Threats J.* 2013;6. 10.3402/ehtj.v6i0.19958 23362417 PMC3557954

[13] Olson DH Aanensen DM Ronnenberg KL: Mapping the global emergence of *Batrachochytrium dendrobatidis*, the amphibian chytrid fungus. *PLoS One.* 2013;8(2):e56802. 10.1371/journal.pone.0056802 23463502 PMC3584086

[14] The New Indian Express: Mission Rabies or mission Impossible? Reference Source

[15] Food and Agriculture Organization of the United Nations (FAO) 2013 – Cell phones revolutionizing Kenya’s livestock sector. Reference Source

[16] The Schistosomiasis Consortium for Operational Research and Evaluation. Reference Source

[17] The Schistosomiasis Control Initiative. Reference Source

[18] National Public Radio Dec 2012 – The sight of Roadkill makes a pretty Data-Rich picture. Reference Source

[19] W3C HTML5 Specification. Reference Source

[20] Aanensen DM Huntley DM Menegazzo M: F1000Research/EpiCollectplus. *Zenodo.* 2014 Data Source 10.12688/f1000research.4702.1 PMC4244764 25485096

[21] Aanensen DM Huntley DM Menegazzo M: F1000Research/EcPlusAndroid. *Zenodo.* 2014 Data Source 10.12688/f1000research.4702.1 PMC4244764 25485096

