# Supplementary material for: EpiCollect+: linking smartphones to web applications for complex data collection projects
Source: F1000Res. 2014 Aug 20;3:199. [Version 1] doi: 10.12688/f1000research.4702.1 (PMC4244764; doi:10.12688/f1000research.4702.1)
Supplement: Peer review history

# Peer review: Referee response for version 1

**DOI:** 10.5256/f1000research.5024.r5899

**Published:** 2014-11-17

**Authors:** Jason Stajich

**Affiliations:** 1 Department of Plant Pathology & Microbiology, University of California, Riverside, USA

This manuscript provides a description of the EpiCollect+ tool for smartphone based collection of data for web applications. It describes open source software for the Android platform to build up data collection sources and integration into databases.

I have read this submission. I believe that I have an appropriate level of expertise to confirm that it is of an acceptable scientific standard.

# Peer review: Referee response for version 1

**DOI:** 10.5256/f1000research.5024.r6371

**Published:** 2014-10-22

**Authors:** Daniel Lombraña González

**Affiliations:** 1 Crowdcrafting, Madrid, Spain

This paper describes properly the development and features of the software EpiCollect and its new version EpiCollect+.

The paper describes in detail how the software works, giving a proper step by step tutorial about how to create a project, use the project, and see the charts and maps related to the collected data.

As a suggestion for improving the paper, I would say that:

- A table comparison between different frameworks/solutions in order to have a clear idea about what makes EpiCollect+ different from other solutions.
- The paper should address a bit more of the new features of its new HTML5 version, giving a bit more of information about its beta version.

In general I recommend this paper to be accepted and indexed.

I have read this submission. I believe that I have an appropriate level of expertise to confirm that it is of an acceptable scientific standard.
